# Consciousness in Neurocritical Care Cohort Study Using fMRI and EEG (CONNECT-ME): Protocol for a Longitudinal Prospective Study and a Tertiary Clinical Care Service

**DOI:** 10.3389/fneur.2018.01012

**Published:** 2018-11-27

**Authors:** Anine P. Skibsted, Moshgan Amiri, Patrick M. Fisher, Annette Sidaros, Melita Cacic Hribljan, Vibeke Andrée Larsen, Joan Lilja S. Højgaard, Miki Nikolic, John Hauerberg, Martin E. Fabricius, Gitte Moos Knudsen, Kirsten Møller, Daniel Kondziella

**Affiliations:** ^1^Department of Neurology, Rigshospitalet, Copenhagen University Hospital, Copenhagen, Denmark; ^2^Neurobiology Research Unit, Copenhagen University Hospital and Center for Integrated Molecular Brain Imaging, Copenhagen, Denmark; ^3^Department of Clinical Neurophysiology, Rigshospitalet, Copenhagen University Hospital, Copenhagen, Denmark; ^4^Department of Radiology, Rigshospitalet, Copenhagen University Hospital, Copenhagen, Denmark; ^5^Department of Neurosurgery, Rigshospitalet, Copenhagen University Hospital, Copenhagen, Denmark; ^6^Faculty of Health and Medical Sciences, University of Copenhagen, Copenhagen, Denmark; ^7^Department of Neuroanaesthesiology, Rigshospitalet, Copenhagen University Hospital, Copenhagen, Denmark

**Keywords:** coma, consciousness, electroencephalography, functional magnetic resonance imaging, locked-in syndrome, magnetic resonance imaging, unresponsive wakefulness syndrome, vegetative state

## Abstract

**Aims and Objectives:** To facilitate individualized assessment of unresponsive patients in the intensive care unit for signs of preserved consciousness after acute brain injury.

**Background:** Physicians and neuroscientists are increasingly recognizing a disturbing dilemma: Brain-injured patients who appear entirely unresponsive at the bedside may show signs of covert consciousness when examined by functional MRI (fMRI) or electroencephalography (EEG). According to a recent meta-analysis, roughly 15% of behaviorally unresponsive brain-injured patients can participate in mental tasks by modifying their brain activity during EEG- or fMRI-based paradigms, suggesting that they are conscious and misdiagnosed. This has major ethical and practical implications, including prognosis, treatment, resource allocation, and end-of-life decisions. However, EEG- or fMRI-based paradigms have so far typically been tested in chronic brain injury. Hence, as a novel approach, CONNECT-ME will import the full range of consciousness paradigms into neurocritical care.

**Methods:** We will assess intensive care patients with acute brain injury for preserved consciousness by serial and multimodal evaluation using active, passive and resting state fMRI and EEG paradigms, as well as state-of-the-art clinical techniques including pupillometry and sophisticated clinical rating scales such as the Coma Recovery Scale-Revised. In addition, we are establishing a biobank (blood, cerebrospinal fluid and brain tissue, where available) to facilitate future genomic and microbiomic research to search for signatures of consciousness recovery.

**Discussion:** We anticipate that this multimodal approach will add vital clinical information, including detection of preserved consciousness in patients previously thought of as unconscious, and improved (i.e., personalized) prognostication of individual patients. Our aim is two-fold: We wish to establish a cutting-edge tertiary care clinical service for unresponsive patients in the intensive care unit and lay the foundation for a fruitful multidisciplinary research environment for the study of consciousness in acute brain injury. Of note, CONNECT-ME will not only enhance our understanding of consciousness disorders in acute brain injury but it will also raise awareness for these patients who, for obvious reasons, have lacked a voice so far.

**Trial registration:** The study is registered with clinicaltrials.org (ClinicalTrials.gov Identifier: NCT02644265).

## Introduction

**Figure 1:**
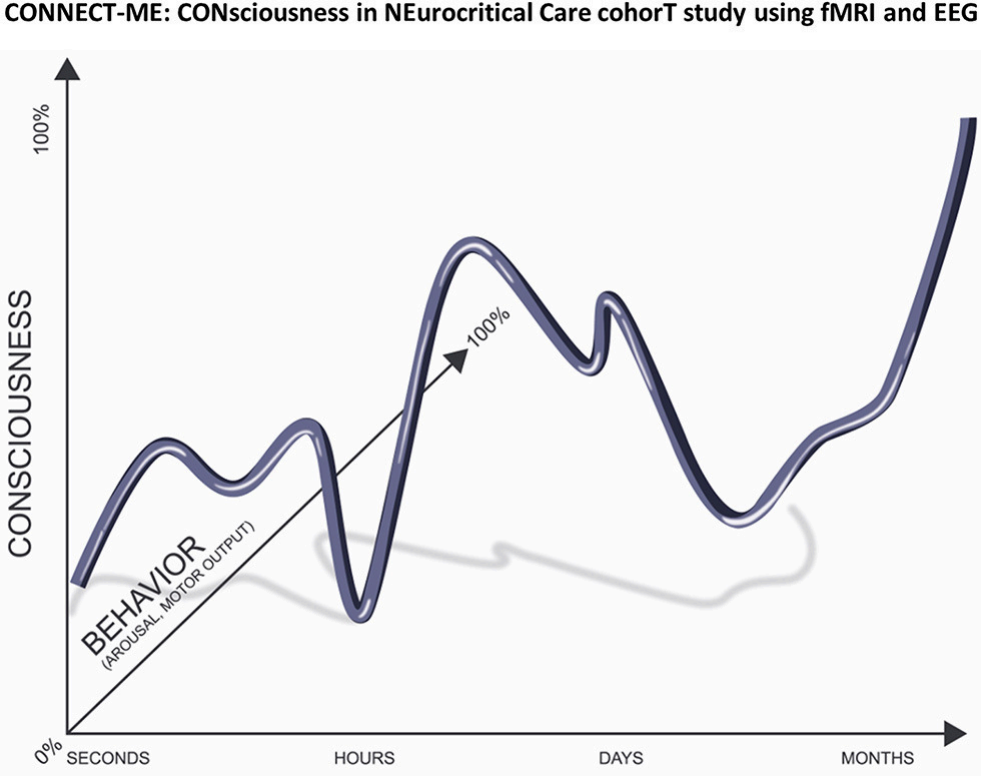
Correct evaluation of consciousness depends on the patient's behavior, which can be conceptualized as a product of arousal and motor output. Many patients with acute brain injury will only be aroused by certain stimuli and they lack motor output because of paralysis. In addition, their level of consciousness typically fluctuates with time. Thus, these patients are often wrongly believed to be unconscious. Our project CONNECT-ME will therefore employ serial and multimodal evaluations including state-of-the art fMRI and EEG technology in order detect covert consciousness in patients with acute brain injury. Adapted with permission from Kondziella et al. (1).

Searching for consciousness in patients with acute brain injury by means of clinical examination is difficult because patients must be awake, they must possess the voluntary drive to mobilize motor function, and this motor function must be preserved to a degree that is easily measurable. Moreover, consciousness levels fluctuate, and all these requirements need to be fulfilled at the time of examination (1–5) (Figure 1). Thus, many patients with disorders of consciousness (DoC) are incorrectly classified as being in a vegetative state (VS) (5). This has major ethical and practical implications for patients and their caregivers, including prognosis, treatment, resource allocation, and end-of-life decisions (6–10). For instance, most deaths (~70%) in the neuro-intensive care unit (ICU) occur following a decision to withdraw life-sustaining therapy (11), but the accuracy of current prognostic indicators and the evidence from controlled studies to guide decision-making are limited. This leads to a high risk of ‘self-fulfilling prophecy', i.e., misclassification of consciousness levels and premature prognostication, resulting in early withdrawal of life-sustaining therapy and death (12).

In order to circumvent the need for motor function, consciousness paradigms using functional magnetic resonance imaging (fMRI) and electroencephalography (EEG) have been developed (13–15). An estimated 15% of patients with a clinical diagnosis of VS can follow commands by performing mental imagery tasks when examined with fMRI and/or EEG paradigms, strongly suggesting that they are indeed conscious—at least every now and then (1). Accordingly, new concepts have emerged that defy established neurological practice such as the concept of cognitive motor dissociation (i.e., command following during fMRI and EEG despite being unresponsive at the bedside) (16). However, EEG- and fMRI-based paradigms have so far typically been tested in patients with chronic brain injury only, and the incidence of cognitive motor dissociation in the neuro-ICU is unknown (16).

Therefore, as a novel approach, *CONNECT-ME: Consciousness in neurocritical care cohort study using fMRI and EEG* (ClinicalTrials.gov Identifier: NCT02644265) is designed to import the full range of consciousness paradigms into neurocritical care. We aim to assess patients with acute brain injury for preserved consciousness by serial multimodal evaluations using active, passive and resting state fMRI- and EEG-based paradigms, as well as state-of-the art clinical rating scales and sophisticated bedside techniques such as pupillometry. Moreover, we are establishing a biobank (blood, cerebrospinal fluid, and feces) for genomic, metabolomic, and microbiomic research. All technological and scientific expertise is in place, including guidance by an International Scientific Advisory Board.

We anticipate that this approach will add essential clinical information, including detection of preserved consciousness in patients previously thought of as unconscious. The ability to identify preserved cognitive abilities following acute brain injury is of utmost importance to enable personalized diagnostics, to guide therapeutic decisions and to better predict outcome in non-responsive patients. This also represents an intriguing opportunity to institute focused rehabilitation early after the injury and to use rehabilitation resources in the most efficient way (17, 18).

The aim of CONNECT-ME is therefore two-fold: We wish to lay the foundation for a fruitful multidisciplinary research environment for the study of consciousness in acute brain injury, and at the same time, we will establish a cutting-edge tertiary care clinical service for unresponsive patients in the intensive care unit.

## Methods and design

The present protocol follows the requirements of the SPIRIT checklist (19).

### Research questions and objectives

We aim to establish, validate and improve fMRI- and EEG-based consciousness paradigms, as well as sophisticated clinical rating scales and bedside techniques such as pupillometry, for patients with acute brain injury in the ICU and step-down units. We will achieve this by using a multidisciplinary approach including expertise from neurology, neurosurgery clinical neurophysiology, anesthesiology, and functional neuroimaging. We hypothesize that serial multimodal assessments better reflect changing levels of consciousness than single unimodal evaluations. In addition, we wish to establish a biobank (blood, cerebrospinal fluid, and feces) for future genomic, metabolomic, and microbiomic research. Within 5 years, we will establish a full clinical service and a fruitful research milieu covering the entire spectrum of fMRI- and EEG-based consciousness paradigms in acute brain injury. Eventually, the present research project will lead to more efficient decision making in neurocritical care, thereby optimizing resource allocation and improving quality of life in survivors with decreased motor function because of acute brain injury.

#### Primary objectives

We aim to rigorously and systematically examine non-communicating patients with acute brain injury for preserved consciousness. To this end, we will set up fMRI- and EEG-based consciousness paradigms, implement standardized clinical rating scales into daily neurological routine and collect all relevant data in a prospective longitudinal database. Specifically, we wish to:

- Establish and validate fMRI- and EEG-based consciousness paradigms in the ICU as well as in neurological and neurosurgical step-down units;
- Establish a clinical service for assessment of covert consciousness and cognitive abilities in non-communicating patients with acute brain injury who have lost motor output;
- Establish a biobank (blood, cerebrospinal fluid, and feces) for genomic, metabolomic, and microbiomic research;
- Establish a fruitful research environment on fMRI- and EEG-based consciousness paradigms in acute brain injury.

#### Secondary objectives

We wish to pool fMRI- and EEG-based data as well as results from systematic clinical evaluations and genomic and metabolomic data to study the neuronal mechanisms by which patients regain consciousness following acute brain injury.

We will test the following hypotheses:

- fMRI- and EEG-based consciousness paradigms are feasible in patients with complete (or near-complete) loss of motor function in the acute phase of brain injury.
- fMRI- and EEG-based consciousness paradigms, including state-of-the-art clinical rating scales and bedside techniques such as pupillometry, applied in the acute phase of brain injury, will add clinically relevant information that complements standard bedside examination. This includes detection of preserved consciousness in some patients who were clinically classified as being unconscious due to lost motor output.
- fMRI- and EEG-based consciousness paradigms do not always yield the same evidence for the presence or absence of consciousness in acute brain injury.
- Serial multimodal evaluations will better reflect a changing level of consciousness than single unimodal assessments.
- In most patients who regain consciousness after acute brain injury, the following pattern will be observed: Intrinsic cortical connectivity, including fronto-parietal networks, as revealed by resting state paradigms will recover first, followed by the occurrence of cognitive evoked potentials and cortical activation (passive paradigms), and lastly, the ability to follow commands (active paradigms).
- Because of the absence of a gold standard for consciousness (1), a composite reference standard, considering bedside examination, clinical rating scales and fMRI- and EEG-based consciousness paradigms, allows the most reliable classification of patients with DoC (16).
- Genomic and metabolomic data may reveal signatures for consciousness recovery.

### Target condition, outcome measures, inclusion, and exclusion criteria

We define the target condition (*primary outcome*) as signs of preserved consciousness in non-communicating patients with DoC due to traumatic brain injury (TBI) or non-traumatic TBI, including (but not limited to) cerebrovascular disorders (CVA; including ischemic and hemorrhagic stroke, subarachnoid hemorrhage, and cerebral venous sinus thrombosis), anoxic-ischemic encephalopathy (e.g., due to cardiac arrest), and neuroinflammatory conditions (e.g., autoimmune encephalitides, neuroinfectious disease). The target condition will be assessed using clinical evaluation as well as fMRI- and EEG-based consciousness paradigms (*outcome measures*), including command following (active paradigms), preserved cognitive evoked potentials or cortical activation (passive paradigms; fMRI, respectively, EEG), and intrinsic functional connectivity, including the auditory and default mode networks (resting state paradigms). Results from fMRI- and EEG-based consciousness paradigms will be analyzed for similarities and dissimilarities and correlated with clinical data from standardized bedside examination and clinical rating scales to learn about the neuronal mechanisms by which patients recover consciousness following acute brain injury (*secondary outcomes*).

#### Target population

Our target population consists of adult non-communicating patients with DoC due to TBI and non-TBI (age >15 years). We apply the classical definition of consciousness as a “state of full awareness of the self and one's relationship to the environment” (20).

#### Inclusion criteria

Inclusion criteria include non-responding patients (clinically defined as coma, VS/unresponsive wakefulness syndrome (UWS), minimal conscious state (MCS), emerged from MCS (eMCS), or locked-in syndrome) with acute or sub-acute (arbitrarily defined as ≤ 31 days from injury) TBI or non-TBI, needing structural MRI for clinical reasons (diagnosis, prognostication). The great majority of these patients will be intubated on ventilatory support. We will aim for unsedated patients. However, if patients cannot be weaned from sedation, the level of sedation will be lowered to the lowest possible level.

#### Exclusion criteria

Exclusion criteria include contraindications for examination by MRI, severe cardiorespiratory compromise and similar acutely life-threatening conditions, evidence of severe pre-morbid neurological deficits such as aphasia or deafness, lack of Danish or English language proficiency, age <15 years, and evidence of defect auditory and sensory pathways [if clinically suspected or as revealed by pretest screening with brainstem auditory evoked potentials (BAEP) and somatosensory evoked potentials (SSEP)]. Sedation is an important confounder during fMRI scans that must be carefully monitored; we will therefore also exclude patients requiring sedation levels exceeding 1 mg/kg/h propofol at baseline. Although we will aim for no sedation or the lowest possible level of sedation to reduce movement artifacts, we will leave the choice of the sedative drug and the dosage to the discretion of the responsible anesthetist. [Fortunately, recent studies indicate that connectivity decreases associated with propofol and sevoflurane sedation, involving the thalamus and insula, are relatively small compared with those already caused by structural brain injury (21, 22)].

#### Definitions

The term DoC includes patients in coma, VS/UWS, and MCS, as well as those who have emerged—but not completely—from MCS (eMCS). *Coma* may be defined as a state of profound unawareness from which patients cannot be aroused. Crucially, a normal sleep-wake cycle is absent. This lasts usually only a few days to 3 weeks following acute brain injury. In contrast, the *VS/UWS*, is a condition of wakefulness without awareness (4). Patients in this condition may open their eyes but exhibit only reflex behaviors and are therefore considered unaware of themselves and their surroundings. In comparison, patients in *MCS* show unequivocal signs of non-reflex behaviors occurring inconsistently, yet reproducibly, in response to environmental stimuli. Although some may follow commands to a certain degree, accurate communication is not possible. VS/UWS and MCS most likely exist on a spectrum rather than being categorically distinct (23, 24). Thus, patients may be classified into MCS plus (i.e., if they are able to obey commands) or minus (i.e., if they only localize pain, exhibit visual pursuit or show appropriate emotional expressions) (25). Traditionally, VS/UWS has been considered permanent 3 months after non-traumatic injuries and 12 months following TBI but late recovery is increasingly recognized (4). [A recent guideline committee therefore suggested that the term ”permanent” should be abandoned (26)]. Patients may evolve from VS/UWS into MCS (or better) and they may or may not relapse. It follows that signs of preserved consciousness can wax and wane (Figure 1).

#### Neurological bedside examination and clinical rating scales

Patients will be assessed using a detailed neurological bedside exam performed by or supervised by board-certified neurologists with experience in neurocritical care. Patients will be examined by standardized clinical rating scales, including (but not limited to) Glasgow Coma Scale (GCS), Full Outline of UnResponsiveness (FOUR), and Coma Recovery Scale-Revised (CRS-R) (27). Examinations will be performed daily on the ward as well as directly (within 30 min) prior and after each fMRI and EEG session.

#### fMRI- and EEG-based consciousness paradigms (active, passive, and resting state)

Whereas, active paradigms of consciousness may suggest a higher degree of certainty, passive, and resting state paradigms should also allow detecting signs of consciousness in patients who are not able to cooperate in cognitive tasks because of aphasia, neglect, executive dysfunction, major depression or deafness (1). Indeed, in a recent landmark study, it was shown that intrinsic functional connectivity assessed by fMRI, including (but not limited to) the auditory and default mode networks, may differentiate MCS from VS/UWS at the single-patient level (28). Thus, as stated above, we will establish the full range of fMRI- and EEG-based paradigms, including active, passive, and resting state paradigms.

### Study design and setting

**Figure 2:**
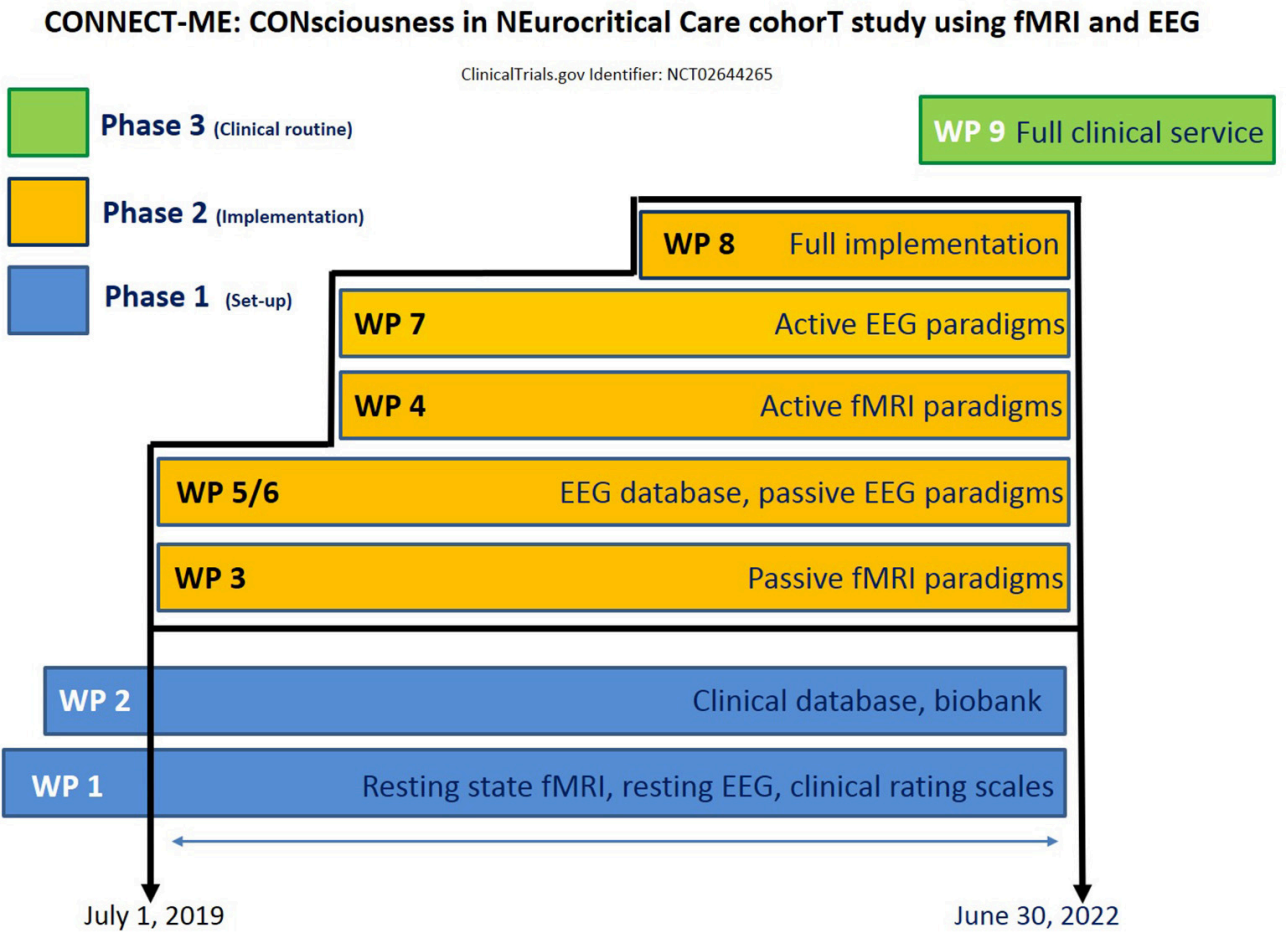
Schematic overview of the work packages and timeline of the project.

The study will be performed at the Neurocentret, Rigshospitalet, Copenhagen University Hospital, with input from the departments of neurology, neurosurgery, clinical neurophysiology, neuroanesthesiology, and radiology, as well as the Neurobiology Research Unit (University of Copenhagen). To facilitate this complex project in the most efficient way, we have divided it into 9 work packages (Figure 2). Work packages 1–2 cover the first phase of the project (set-up), work package 3–8 covers the second phase (clinical implementation) and work package 9 covers the third phase (clinical routine). The vision is that once work package 9 has started, CONNECT-ME will transform into a fully functional tertiary care patient service and enable ongoing prospective data acquisition for clinical research purposes.

#### Work package 1 (resting state fMRI; resting state EEG, clinical protocol)

Enrollment began on April 12, 2017 (24 patients have been enrolled as of September 1, 2018). We are evaluating a convenience sample of DoC patients with acute brain injury (*n* = 24), admitted to the ICU and/or neurological and neurosurgical step-down units, using resting state fMRI and EEG. Relevant protocols are established at our institution. Pilot data from a previous cohort on resting state fMRI have been published (21).

##### fMRI

Resting-state fMRI are performed on a 3T Prisma MRI scanner with 64-channel head coil. Despite altered states of consciousness, participants are instructed to close their eyes and let their mind wander, but not to fall asleep. During the procedure, patients are monitored by an experienced neuroanesthesiologist and a nurse anesthetist; patients are sedated, if necessary, with sevoflurane or propofol at low doses for optimal image acquisition and mechanically ventilated aiming at normal ventilation. Participants complete a 10-min T2^*^-weighted echo-planar imaging BOLD fMRI sequence (no. of volumes = 300 volumes, TR/TE = 2,000/30 ms, flip angle = 90°, in-plane matrix 64 × 64, number of slices = 32, voxel size = 3.6 × 3.6 × 3.75 mm). A high-resolution 3D T1- weighted structural image is acquired using a sagittal, magnetization prepared rapid gradient echo (MP-RAGE) sequence (TR/TE/TI = 1,900/2.58/900 ms, flip angle = 9°, in-plane matrix 256 × 256, number of slices = 224, voxel size = 0.9 × 0.9 × 0.9 mm). We use single-subject seed-based analysis to estimate seed-to-voxel and seed-to-seed connectivity. Functional imaging data are pre-processed using SPM12 (https://www.fil.ion.ucl.ac.uk/spm/software/spm12/) and Conn v16.b (29). We will visually inspect all acquired scans to assess data quality and document morphological abnormalities that may introduce noise. We account for physiological and other noise sources using methods typically applied to resting-state fMRI data. This includes applying a bandpass filter (range 0.008–0.09 Hz) and denoising gray-matter voxel time series using aCompCor (30). We will quantify default-mode network and other canonical resting-state networks using regions defined from an independent dataset (e.g., “networks” region set in Conn). We use Fisher's r-to-z transformation to convert the estimated correlation coefficient (rho) between any of two means, denoised time series. Qualitative assessment of the presence of DMN (21) and other networks will be complemented by data-driven strategies, including defining deviation from normal DMN connectivity defined as a multivariate distribution based on a reference healthy control population that has been scanned on the same MRI using scan parameters identical to those we use here (31). Investigators classifying the DMN are blinded to clinical outcome.

##### EEG

EEG will be recorded using standard clinical procedures including a 28-channel system and video observations within 1 h before or after fMRI (or as close to this time as possible) and read by board-certified electroencephalographers with experience in neurocritical care EEG. Artifacts such as those due to excessive muscular activity will be dealt with according to standard clinical procedures (e.g., adjustment of EEG equipment, removing sources of noise from the environment, post-processual data cleansing). We will assess EEG background activity and EEG reactivity both by visual and by spectral EEG analysis, including absolute and relative EEG power in different frequency bands. *EEG background activity:* Ten minutes of resting state EEG with the patient in a quiet environment will be analyzed; artifacts in the ICU settings such as background noise will be dealt with and prevented (if possible) according to standard clinical procedures. In addition to visual EEG analysis by board-certified electroencephalographers, 2x6 10 s artifact-free EEG segments will be compared pairwise and analyzed using EEG spectral analysis to assess for spontaneous fluctuations in the background activity (which allows us to distinguish between spontaneous fluctuations and evoked reactive EEG changes following external stimuli during subsequent reactivity testing). *Reactivity:* EEG reactivity is defined as any change in frequency or amplitude of the EEG background activity resulting from a specific external stimulus (32). However, although different methods exist to assess EEG reactivity, all those methods are poorly validated (or not at all). In a recent review, Azabou et al. analyzed more than 200 studies and concluded that current assessment methods are very heterogeneous, and that consensus for standardizations of stimulations and interpretations is absent (33). Therefore, we will use a battery of easy-to-implement stimulations and evaluate EEG reactivity both visually and using spectral EEG analysis, which will reduce interrater variability. To test EEG reactivity, we will apply auditory, sensory and nociceptive stimuli as well as eye opening. Each stimulation will be analyzed and graded for presence or absence of reactivity in the EEG background. Auditory stimulation by calling the patient by their own name has been shown to elicit a robust electrophysiological response, especially when involving a familiar voice (34). Further, we will apply nociceptive (10 s of compression of the earlobes bilaterally), sensory (light touch involving the external openings of the nostrils) and visual stimulation (passive eye opening/eye closure). All procedures will be performed by trained EEG technicians supervised by the CONNECT-ME study team. Absolute total and relative power will be evaluated in the delta (1–4 Hz), theta (4–8 Hz), alpha (8–13 Hz), beta (13–30 Hz), and gamma (>30Hz) bands and averaged within each EEG channel. For detailed analysis the electrodes will be grouped into four regions of interest: fronto-central (Fp1, Fp2, F3, F4, C3, C4), temporal (F7, F8, T7, T8, P7, P8), parieto-occipital (P3, P4, O1, O2), and midline (Fz, Cz, Pz). EEG patterns will be classified according to Synek's scale (35). In addition to conventional analyses and once a sufficient number of patients has been included (~from 50 patients onwards), we will aim for data-driven approaches as well, using machine learning paradigms, to search for EEG markers of consciousness such as recently proposed (36–38).

##### Clinical rating scales

At the same time, we are establishing a systematic clinical examination protocol, including, but not limited to, the FOUR and CRS-R, and sophisticated novel bedside techniques such as pupillometry. Automated pupillometry will be used both for quantitative measurement of pupillary indices [e.g., pupillary diameter, constriction, and dilation velocities, percentage change in pupillary size following light stimulation (39)] and for assessment of command following using a mental arithmetic paradigm [a modified version of (40)] from Using the CRS-R (41), patients are being diagnosed as in a VS/UWS according to the following criteria [The Multi-Society Task Force on PVS, 1994; adapted from Demertzi et al. (28)]: (A) Patients show no evidence of awareness of self or the environment and are not able to interact with others. (B) They show no evidence of sustained, reproducible, purposeful, or voluntary behavioral responses to auditory, tactile, visual, or noxious stimuli (C) They lack evidence of language comprehension or expression. (D) Patients have alternating periods with open and closed eyes. (E) Hypothalamic and brainstem autonomic functions are sufficiently preserved to permit survival with medical and nursing care. (F) There is bowel and bladder incontinence. (G) Cranial-nerve and spinal reflexes are variably preserved. In contrast, patients will be diagnosed as in MCS when they demonstrate discernible evidence of awareness of self or environment, on a reproducible or sustained basis, by at least one of the following behaviors (27, 42, 43): (A) Purposeful behavior (including movements or affective behavior occurring in relation to relevant environment stimuli and not due to reflexive activity), such as visual pursuit or sustained fixation occurring in direct response to moving or salient stimuli, smiling or crying in response to verbal or visual emotional (but not neutral) stimuli, reaching for objects demonstrating a relationship between object location and direction of reach, touching or holding objects in a manner that accommodates the size and shape of the object, vocalizations or gestures occurring in direct response to the linguistic content of questions. (B) Command following. (C) Gestural or verbal yes/no response (regardless of accuracy). (D). Intelligible verbalization. Emergence from the MCS is defined by the return of functional communication and/or object use. In addition to CRS-R, we are examining patients using the GCS and FOUR (44), as well as comprehensive clinical examinations tailored to the specific patient case at hand and supervised by a board-certified neurologist (DK) with more than 8 years' experience in neurocritical care.

#### Work package 2 (clinical database, biobank)

To maximize the learning effect and to facilitate research, we will collect all relevant clinical, neurophysiological and imaging data in a comprehensive longitudinal database. This database is currently being developed, using REDCap which is provided free of charge to health care providers within the Capital Region of Denmark (45). At present, the database consists of 11 instruments (*Study ID; Cause of ICU admission; Baseline Data at ICU admission; Laboratory investigations; Neurological exam; MRI, including fMRI; EEG; Pupillometry; Neurological exam and data at ICU discharge; 3 months follow-up; and 12 months follow-up*) and at total of 555 fields (= questions). For a given patient, only a subset of these fields needs to be answered, depending on the diagnosis at admission and initial diagnostic procedures. Clinical outcome data will be assessed by a trained research nurse and a clinical PhD student, either by telephone interview or during follow up visits, using established rating scales (e.g., Coma Recovery Scale-Revised, modified Rankin Scale, Barthel index) at hospital discharge and at 3, respectively, 12 months. Target clinical data include (but are not limited to): age, sex, previous medical history (including hereditary predispositions), medication (including drugs with potential influence on consciousness levels such as antiepileptics or antidepressants), cause of admission, sedation, surgical and non-surgical complications, comprehensive laboratory investigations, neurological examination, CRS-R, GCS, FOUR, and outcome data including modified Rankin Scale (mRS), Barthel index, Glasgow Outcome Scale (GOS), as well as whether at follow-up the patient lives at home, is admitted to a rehabilitation facility, or resides in a nursing home. Neurophysiological data include (but are not limited to) resting state, passive and active EEG paradigms and (where appropriate) evoked potentials, including SSEP and BAEP. Imaging data include data from resting state, passive and active fMRI paradigms and structural MRI, including (but not limited to) T1- and T2-weighted imaging, DWI, FLAIR, gradient echo sequences and (where appropriate) MR spectroscopy and tractography. In addition, we are setting up a biobank for cerebrospinal fluid, blood and feces samples for future studies related to genomics, metabolomics and microbiomics. Minus 80° freezer facilities are available at our institution.

#### Work package 3 (passive fMRI paradigms)

We will set-up a passive fMRI paradigm using two oddball paradigms (“subject's own name” and semantic ambiguity), and we will assess patients (*n* = 12) clinically as outlined above. Active and passive paradigms will be set-up together with fMRI engineers and data analysists from the Neurobiology Research Unit, University of Copenhagen. Prior to inclusion, patients may be examined by brainstem auditory evoked potentials (BAEP) and somatosensory evoked potentials (SSEP) to ensure intact primary auditory and sensory cortex function.

#### Work package 4 (active fMRI paradigms)

We will establish an active fMRI paradigm by means of visual imagery tasks (playing tennis, navigating in a familiar surrounding) as described earlier [for review, see (1)] and a novel motor imagery task (finger tapping; opening and closing a fist) developed in-house, using a similar convenience sample (*n* = 12) as in work package 1. Patients will be clinically evaluated on a daily basis; including 60 min prior to and after each fMRI assessment, in order to capture fluctuations in consciousness levels as accurately as possible.

#### Work package 5 (clinical EEG database)

In order to correlate resting state EEG with clinical outcome data, we will assess a clinical EEG database, available at the Department of Clinical Neurophysiology, Rigshospitalet, Copenhagen University Hospital, for EEG complexity and other advanced EEG measures following acute brain injury [for review, see (1)].

#### Work package 6 (passive EEG paradigms)

We will set up passive EEG paradigms, using series of standardized behavioral testing, including central pain stimulation, sensory stimulation of the nostrils using a swab, passive eye opening, and oddball paradigms (“subject's own name,” semantic ambiguity) as described previously [for review, see (1, 32, 33, 36, 38)]. For semantic ambiguity and subject's own names, in addition to the markers of EEG complexity outlined earlier, we will aim for analysis of evoked potentials, including, but not necessarily limited to, the mismatch negativity (MMN), P300 and N400. However, as the sensitivity of the latter has been questioned recently (46), we may opt to adopt alternative EEG markers and evoked potentials as soon as they become validated in the future). Clinical evaluation of patients (*n* = 12) will be performed as described. Healthy volunteers recruited from the staff of our institution will serve as control (*n* = 12). Active and passive EEG paradigms will be set-up together with electrophysiological engineers and data analysists from the Department of Clinical Neurophysiology, Rigshospitalet, Copenhagen University Hospital. As stated earlier, patients may be examined by brainstem auditory evoked potentials (BAEP) and somatosensory evoked potentials (SSEP) to confirm primary auditory and sensory cortex integrity.

#### Work package 7 (active EEG paradigms)

Akin to the fMRI paradigm, we will establish an active EEG paradigm using motor imagery tasks (finger tapping; opening and closing a fist), as described earlier [see work package 6 for analysis of EEG markers and evoked potentials, and for review, see (1, 32, 33, 36, 38)]. Clinical evaluation of patients (*n* = 12) will be performed as outlined. Healthy volunteers recruited from the staff of our institution will serve as control (*n* = 12).

#### Work package 8 (consecutive sample assessed by full range of fMRI- and EEG-paradigms)

In this work package, we wish to combine all consciousness measures in order to systematically and comprehensively evaluate consciousness in each acute brain injury patient, using the full range of clinical assessments as well as active, passive and resting state fMRI- and EEG-based paradigms. We aim for 24 consecutive TBI and/or non-TBI non-communicating DoC patients admitted to our neurological and neurosurgical ICU or step-down units (inclusion criteria). Prior to inclusion, primary auditory and sensory cortex integrity will be verified using BAEP and SSEP. Exclusion criteria will include those referred to in work package 1.

#### Work package 9 (full clinical service)

Once we have shown that comprehensive fMRI- and EEG-based consciousness paradigms are feasible in patients with acute brain injury in the ICU and intermediate care units, we wish to establish a full clinical service and a national referral center for the evaluation of DoC patients following acute brain injury. This will lay the foundation for a fruitful research environment (phase 3).

If necessary, amendments to the work packages will be published on clinicaltrials.org (NCT02644265).

### Statistical analysis

A power calculation of the required sample size that would include the combined different methods does not appear feasible owing to the multimodal design of CONNECT-ME but the numbers of patients recruited into the different work packages reflects empirical numbers derived from previous results on consciousness paradigms in the critical care setting (21, 47). In addition to descriptive statistics, we will perform correlation analysis between behavioral scales (CRS-R total score) and default mode networks, respectively, EEG entropy using Spearman testing. Non-parametric tests will be used for univariate analysis (Wilcoxon rank sum/Mann-Whitney test and Kruskal-Wallis test) to test whether default mode and other brain networks or EEG entropy differ between diagnostic groups (VS/UWS vs. MCS). Machine learning models will be applied to evaluate resting-state fMRI and other neuroimaging measures as predictors of covert consciousness and subsequent recovery (i.e., does imaging data inform patient level of consciousness and/or prognosis). Receiver operating characteristic (ROC) analysis may be used to identify a cut-off value differentiating conscious (MCS) from unconscious (coma or VS/UWS) patients. Results will be considered significant at *p* < 0.05 or lower, if appropriate (e.g., <0.001 for fMRI data). Statistical advice and supervision, including exploration with advanced machine learning algorithms, will be provided in-house (Department of Biomedical Statistics, University of Copenhagen).

### Data management, ethics approval, and patient consent

Patients enrolled into CONNECT-ME are unconscious and per definition unable to consent themselves for the study; therefore, consent will be obtained from their legally authorized representative. Patients recovering consciousness will be asked for re-consent as soon as feasible. Privacy and confidentiality is of utmost importance and data will be de-identified wherever possible. All procedures will be performed according to the Declaration of Helsinki. Approval has been obtained from the Ethics Committee of the Capital Region of Denmark (journal-nr: H-16040845) and by the Danish Data Protection Agency (RH-2016-191, I-Suite nr: 04760). As to the establishment of a biobank for future studies related to metabolomics and genomics, we will prevent genetic data from being linked to an individual's identity outside of a restricted access site. Data will be stored electronically using an independent and secure medium.

#### Registration

The study is registered with clinicaltrials.org (ClinicalTrials.gov Identifier: NCT02644265).

#### Dissemination of results and publication policy

We aim to publish results derived from this project in international peer-reviewed scientific journals as well as to disseminate relevant information to the public by local media (e.g., newspapers, TV). We will decide on authorship using consensus and Vancouver criteria at the earliest convenience on each given publication. Whenever applicable, we will allow for data sharing and provide raw data files.

### Organization and stakeholders

#### Study investigators and collaborators

This project is led by DK (Principle Investigator; PI), Department of Neurology, Rigshospitalet, Copenhagen University Hospital, together with representatives from all involved departments: KM, Neuroanaesthesiology; MF, Clinical Neurophysiology; and GMK, Neurobiology Research Unit (NRU) (Co-PI's, all Rigshospitalet, Copenhagen University Hospital). Collaborators include, but are not limited to, the additional members listed as authors of the present protocol.

#### International scientific advisory board members

The members of the International Scientific Advisory Board provide the study investigators with constructive feedback. Board member include Jens Dreier, MD, PhD, scientific director, and professor at the Center for Stroke Research Berlin, Charité, Germany (assists with aspects of *neurocritical care*); Andrea O. Rossetti, MD, PhD, professor and director of the epilepsy unit at Lausanne University Hospital (Lausanne, Switzerland; *EEG*); Steven Laureys, MD, PhD, professor and scientific director of the Coma Science Group at the Neurology Department and Cyclotron Research Centre of the University Hospital and University of Liège, Belgium (*functional imaging*); and Anirban Dutta, PhD; Associate Professor at The State University of New York, Buffalo (Buffalo, NY, USA; *biomedical engineering*).

#### Patient representative

Rikke Schmidt Kjærgaard, PhD, is CEO of Graphicure, a Copenhagen-based data company specializing in visualization and advanced analytics of personal health data, and author of the memoir'The Blink of an Eye. How I Died and Started Living' (Hodder and Stoughton, 2018), following her recovery from sudden bacterial meningitis, multi-organ failure, septic shock, and long-term coma to waking up, paralyzed, only able to communicate through blinking. She will help to disseminate information about CONNECT-ME to the public and contribute to the communication with lay people (e.g., patient organizations).

### Budget, financing plan, and time schedule

All technical equipment (e.g., fMRI, EEG, pupilometer, minus 80° freezer facilities) and neurological, neurophysiological and neuroimaging expertise is in place. The home institution (Rigshospitalet, Copenhagen University Hospital) covers overhead costs. The entire time schedule covers 5 years (2 years for phase 1; 3 years for phase 2; 1 year for phase 3). Additional funding for PhD students is being applied from relevant internal, private, state and university-/hospital-based funds. At the time of writing, phase 1 is being finalized (resting state fMRI and EEG, REDCap-based clinical database; and a biobank; Figure 2). Phase 2 (clinical implementation) is scheduled for July 1, 2019—June 30, 2022).

### Lay project description

Imagine you are hospitalized and conscious but trapped in your own body—unable to move a muscle or blink with your eyes. A nightmare? Yes. But what if no one was aware of it? If your family, friends and doctors thought you didn't know what was happening around you? Research shows this is true for many thousands of patients with brain damage worldwide who are erroneously thought to be in a “vegetative state.” Fortunately, new methods can open the way to the minds of those people. With functional magnetic resonance imaging (fMRI) and electroencephalography (EEG), we can record brain responses without having to look for motor responses. Patients can be asked to think of something and the answer analyzed from their brain activity. When investigated with fMRI or EEG, 15% of patients who appear to be in a vegetative state can follow commands by performing mental tasks. These patients are conscious and misdiagnosed, which is crucial for prognosis, treatment and resource allocation. We wish to establish these methods at Denmark's largest neurocritical care unit, Rigshospitalet, Copenhagen University Hospital, and we anticipate that this will benefit patients from the entire country both in the acute stage and the chronic phase during rehabilitation.

## Discussion

Consciousness paradigms offer exciting opportunities. They improve our understanding of the biological foundations of human consciousness and, even more importantly, they allow us to assess the consciousness levels of patients with brain damage in far greater detail than previously. So far, however, they have been almost exclusively employed in rehabilitation medicine, addressing patients with chronic brain disorders, typically several years following onset of the injury. Moreover, most of these studies have been restricted to spot assessments, not considering that consciousness fluctuates over time. In addition, important methodological issues persist, including uncertainties about the specificity and sensitivity of the different paradigms and about their applicability in brain disorders of various etiologies (1). Lastly, almost all studies until now have employed either fMRI- or EEG-based paradigms, although the two modalities do not necessarily yield identical results in a given patient but rather complement each other. As we have pointed out previously, systematic evaluation of the similarities and differences of these technologies is essential, preferentially by multimodal serial assessments (1). Our group and others have recently established the feasibility of performing fMRI in the neurointensive care unit (21, 47).

The anticipated impact and learning potential of the project is significant in several ways. At the clinical level, CONNECT-ME has distinct practical implications for individual patients. Our recent meta-analysis shows that many thousands of patients worldwide are mistakenly believed to be in a “vegetative state” (1). Fortunately, there is help on the way—new imaging and neurophysiological methods allow insights into the mental world of these patients. The present project is crucially important for unresponsive patients with acute brain injury who, for obvious reasons, have lacked a voice so far. Therefore, we have named it “CONNECT-ME.”

At a more general scientific level, CONNECT-ME addresses fundamental scientific questions about the origin of human consciousness. How consciousness occurs, i.e., what it is that produces the content of our consciousness, belongs to the great existential human questions for which we lack a satisfactory answer, on the same level as the question about the origin of the universe. It is still impossible to define precisely the cerebral regions that are “minimally sufficient and collectively necessary” (48) for consciousness to occur, but by combining cutting-edge clinical, electrophysiological and neuroimaging paradigms we hope that CONNECT-ME will make a humble contribution to solve this question in the not-so-distant future.

A complex project as CONNECT-ME is subject to potential obstacles, which requires careful risk assessment. First, CONNECT-ME is deeply rooted in the clinical hospital setting—the idea is to take advantage of as many routinely collected clinical data as possible (i.e., clinical, neurophysiology, imaging, laboratory, and similar data). Unfortunately, clinicians often find it hard to act as idea generators for applied research due to an ever-increasing administrative burden and the need for more and more efficient working structures. Similarly, scientists may struggle to allocate time for a discourse with clinicians because increasingly fierce competition for limited funding requires instant scientific results. Further strain on local resources and lack of adequate funding might thus be a cause of concern.

Second, reflecting everyday clinical management of critically ill patients, CONNECT-ME will likely be subject to enrollment bias. On one hand, unstable vital functions of ICU patients may compromise their assessment by MRI (and hence their inclusion into CONNECT-ME) and create a bias to include cases with more benign prognosis. On the other hand, MRI is sometimes ordered to better visualize the extent of (usually ischemic or anoxic) brain damage to inform a decision on treatment withdrawal, thereby possibly creating a bias in the opposite direction and toward more sinister prognosis.

Third, it may turn out that only a minor subset of clinically unresponsive patients in the intensive care unit will profit from active EEG- and fMRI paradigms given the need for vigilance and the ability to follow commands required to participate in abstract mental tasks. Still, as shown in a recent paper such cognitive-motor dissociation does occur in the intensive care setting (47), and resting state (and possibly passive) paradigms also reveal essential information although they do not require active patient participation (21).

Fourth, as a truly multidisciplinary project, CONNECT-ME involves colleagues from the entire range of the clinical neurosciences: neurologists, neurosurgeons, neuroanesthesiologists, neuroradiologists, and neuroscientists. CONNECT-ME critically depends on close collaboration between all involved departments. Hence, adequate stakeholder management is essential. CONNECT-ME will only succeed if all team members share the work load and contribute data on an equal basis, and good stakeholder management is mandatory in this regard. Therefore, we have outlined a detailed management model, including a steering committee as outlined above (“Organization and stakeholders”).

Finally, research on unresponsive people with acute brain injury in the intensive care unit also requires ethical considerations, in particular if there is a potential for the detection of covert consciousness. Estimating the degree of consciousness in acutely-brain injured patients as well as their potential for recovering cognitive function is among the most difficult challenges that physicians are facing in the ICU. Indeed, prognostic uncertainty in the acutely comatose patient has been shown to lead to premature withdrawal of life-sustaining therapy (11). Consequently, novel indicators of consciousness and neurological recovery are urgently needed. EEG- and fMRI-based paradigms hold considerable promise because evidence of preserved neural networks or cognitive function may predict good patient outcome. Whereas, EEG can be performed at the bedside and does not expose patients to any substantial risk, conducting fMRI requires transportation to the MR scanner and thus, the patient has to the leave the comparatively safe surroundings of the ICU. In a recent paper on ethical considerations in fMRI research in acutely comatose patients, Weijer et al. have outlined six ethical issues that ought to be addressed by researchers and research ethics committees (49). We will address all of these here.

1. **“[Are] fMRI [and EEG] a therapeutic or non-therapeutic procedure in the study context?”** The first issue addressed by Weijer and co-workers is about the sufficiency of the evidence base and whether or not fMRI can be regarded as a therapeutic procedure (49). Is the belief justified that the use of fMRI in the study may benefit research participants? We will use the data derived from fMRI- and EEG-based paradigms with the greatest caution while the implementation of these paradigms is on-going (work packages 1–8). However, once they have been established as clinical routine (work package 9), fMRI- and EEG-based paradigms will certainly improve diagnosis at the single subject level. In other words, at the beginning the procedures may be considered non-therapeutic; however, over the course of the study we aim for participants to benefit individually from the fMRI and EEG paradigms, and our intention is to establish fMRI as a therapeutic procedure as the present study evolves.
2. **“Have the risks of research participation, including the risks of intrahospital transport, been minimized consistent with sound scientific design?”** Neurophysiological assessment is associated with no particular risk and can be performed at the bedside. fMRI requires transportation of the patient into the MR scanner; however, the fMRI procedure itself is comparable to a routine clinical MRI session in terms of logistics. EEG and fMRI paradigms will be performed only when cardiorespiratory function has been stabilized, sedation has been or can be safely stopped or reduced to a level that is deemed to be consistent with obtaining useful information from the fMRI images; and no other diagnostic or therapeutic procedures are urgently pending. Patient transportation and procedures will be supervised by experienced anesthetists using state-of-the-art equipment. All procedures will be documented in the patient charts.
3. **“Are the risks of non-therapeutic procedures no more than a minor increase above minimal risk?”** Weijler et al. state that the minimal risk threshold only applies in studies in which fMRI is a non-therapeutic procedure. Thus, this issue is relevant only to the first two phases of the present project (work packages 1–8) (49). As outlined above, we will take all necessary precautions to decrease the risk of the transportation. In addition, fMRI scans will be combined with clinically indicated structural scans where feasible.
4. **“Have study participants been selected equitably?”** Participants must be able to tolerate lying flat in the MRI scanner. Patients with high dose sedation, seizure activity or a high risk of serious adverse events will be excluded from study participation.
5. **“Will valid surrogate consent for study participation be obtained?”** Written accept will be obtained from the patient's legal representatives, as the patients are per definition unable to consent themselves for the study at inclusion. If the patient regains consciousness to a sufficient degree, consent will be obtained again. Withdrawal from the study is possible at any given time point without explanation. Withdrawal will not influence diagnostic or therapeutic considerations by the clinical team.
6. **“Are adequate plans in place to share summary and individual research results with the responsible physician or the family?”** A summary of the research results will be shared with the patient or next-of-kin as well as with the treating physicians, if requested (work package 1–8). Once fMRI- and EEG-based paradigms are an established clinical service (work package 9), a detailed account of the results will be made available to patients, the family and all involved physicians on a routine basis.

How will knowledge derived from technology-assisted consciousness evaluation influence our treatment decisions? Concerns may arise primarily around false negative evaluations (i.e., suggesting erroneously that patients are in a VS/UWS, whereas in fact, they are conscious) that may lead to withdrawal of therapeutic resources. However, it should be noted that technology-assisted evaluation merely compliments the bedside evaluation which in itself is flawed. Indeed, as many as 40% of non-communicating patients with DoC are erroneously classified clinically as being in the vegetative state (5). Thus, although we agree that self-fulfilling prophecy must be avoided, the ethical dilemma introduced by technology-assisted evaluation of consciousness reflects already well-known uncertainties in daily decision making in the ICU (49). As is the case for clinically routine diagnostic and prognostic procedures, we will exert the utmost caution when incorporating the results of fMRI- and EEG-based paradigms in prognostic considerations. Indeed, results from these paradigms will only be made available to the attending clinicians after successful completion of the relevant work packages.

## Conclusions

CONNECT-ME aims at improving the diagnosis, prognostication, and care of arguably the most vulnerable patient group that exists, i.e., patients in the intensive care unit who are unresponsive and unable to communicate because of decreased consciousness following acute brain injury. Far too little is understood why some of these patients recover consciousness while others do not. Even more disturbingly, we are beginning to realize that a substantial part of these patients only *appears* to be unconscious—in fact, they are awake and aware, yet incapable to show it because of complete loss of motor function. Unable to speak or move, they are wrongly diagnosed as being in a VS/UWS, facing serious risks including denial of rehabilitation and withdrawal of life-supporting therapy.

Current diagnostic standards are insufficient to identify these patients. What is needed is a sophisticated multimodal and personalized approach, including serial investigations using functional MRI and EEG-based consciousness paradigms, state-of-the-art clinical rating scales and novel non-invasive bedside techniques such as pupillometry, complemented by genomic, and metabolomic data. Such a comprehensive approach has never been established in neurocritical care before. To close this gap, we have initiated CONNECT-ME. We have gathered a multidisciplinary team, dedicated to implement CONNECT-ME at Denmark's largest and most specialized neurocritical care unit. To show the feasibility of our approach, we have published pilot data (10) and a comprehensive meta-analysis summarizing the present state of the field (1). Our goal is to connect to unresponsive patients with acute brain injury who, for evident reasons, have been unable to alert us to their tragic situation so far.

## Author contributions

DK, GK, KM and MF contributed to the conception and design of the study. DK is the Principal Investigator and Project Coordinator and wrote the manuscript. All authors contributed to the intellectual conception, revision of important intellectual content and approval of the final version of this manuscript.

### Conflict of interest statement

The authors declare that the research was conducted in the absence of any commercial or financial relationships that could be construed as a potential conflict of interest.

## Abbreviations

BAEP: Brainstem auditory evoked potentials
BOLD: blood oxygen level dependent
CRS-R: Coma Recovery Scale-Revised
DRS: Disability Rating Scale
DoC: disorders of consciousness
EEG: electroencephalography
eMCS: emerged from minimal conscious state
FLAIR: fluid attenuation inversion recovery
FOUR: Full Outline of UnResponsiveness
fMRI: functional magnetic resonance imaging
GCS: Glasgow Coma Scale
GOS: Glasgow Outcome Scale
ICU: intensive care unit
MRI: magnetic resonance imaging
MCS: minimal conscious state
mRS: modified Rankin Scale
PI: principal investigator
SSEP: somatosensory evoked potentials
SPIRIT: Standard Protocol Items: Recommendations for Interventional Trials
TBI: traumatic brain injury
UWS: unresponsive wakefulness syndrome
VS: vegetative state
WP: work package.

## References

[1] Kondziella D Friberg CK Frokjaer VG Fabricius M Møller K. Preserved consciousness in vegetative and minimal conscious states: systematic review and meta-analysis. J Neurol Neurosurg Psychiatry (2016) 87:485–92. 10.1136/jnnp-2015-310958 26139551

[2] Schnakers C Perrin F Schabus M Hustinx R Majerus S Moonen G. Detecting consciousness in a total locked-in syndrome: an active event-related paradigm. Neurocase (2009) 15:271–7. 10.1080/13554790902724904 19241281

[3] Di H Nie Y Hu X Tong Y Heine L Wannez S. Assessment of visual fixation in vegetative and minimally conscious states. BMC Neurol. (2014) 14:147. 10.1186/1471-2377-14-147 25027769 PMC4112970

[4] Laureys S Celesia GG Cohadon F Lavrijsen J León-Carrión J Sannita WG Unresponsive wakefulness syndrome: a new name for the vegetative state or apallic syndrome. BMC Med. (2010) 8:68 10.1186/1741-7015-8-68 21040571 PMC2987895

[5] Schnakers C Vanhaudenhuyse A Giacino J Ventura M Boly M Majerus S. Diagnostic accuracy of the vegetative and minimally conscious state: clinical consensus versus standardized neurobehavioral assessment. BMC Neurol. (2009) 9:35. 10.1186/1471-2377-9-35 19622138 PMC2718857

[6] Kondziella D Roald Dahl and the complete locked-in syndrome: “Cold dead body, living brain.” J Neurol Sci. (2017) 379:276–8. 10.1016/j.jns.2017.06.033 28716259

[7] Laureys S Pellas F Van Eeckhout P Ghorbel S Schnakers C Perrin F. The locked-in syndrome : what is it like to be conscious but paralyzed and voiceless? Prog Brain Res. (2005) 150:495–511. 10.1016/S0079-6123(05)50034-7 16186044

[8] Di Perri C Bahri MA Amico E Thibaut A Heine L Antonopoulos G. Neural correlates of consciousness in patients who have emerged from a minimally conscious state: a cross-sectional multimodal imaging study. Lancet Neurol. (2016) 15:830–42. 10.1016/S1474-4422(16)00111-3 27131917

[9] Demertzi A Jox RJ Racine E Laureys S. A European survey on attitudes towards pain and end-of-life issues in locked-in syndrome. Brain Inj. (2014) 28:1209–15. 10.3109/02699052.2014.920526 24911332

[10] Kondziella D. Functional neuroimaging in disorders of consciousness: raising awareness for those with decreased awareness. Neuroscience (2018) 382:125–6. 10.1016/j.neuroscience.2018.03.046 29804647

[11] Turgeon AF Lauzier F Simard J-F Scales DC Burns KEA Moore L. Mortality associated with withdrawal of life-sustaining therapy for patients with severe traumatic brain injury: a Canadian multicentre cohort study. CMAJ (2011) 183:1581–8. 10.1503/cmaj.101786 21876014 PMC3185074

[12] Harvey D Butler J Groves J Manara A Menon D Thomas E. Management of perceived devastating brain injury after hospital admission: a consensus statement from stakeholder professional organizations. Br J Anaesth. (2018) 120:138–45. 10.1016/j.bja.2017.10.002 29397121

[13] Stender J Gosseries O Bruno M-A Charland-Verville V Vanhaudenhuyse A Demertzi A. Diagnostic precision of PET imaging and functional MRI in disorders of consciousness: a clinical validation study. Lancet (2014) 384:514–22. 10.1016/S0140-6736(14)60042-8 24746174

[14] Cruse D Chennu S Chatelle C Bekinschtein TA Fernández-Espejo D Pickard JD. Bedside detection of awareness in the vegetative state: a cohort study. Lancet (2011) 378:2088–94. 10.1016/S0140-6736(11)61224-5 22078855

[15] Monti MM Vanhaudenhuyse A Coleman MR Boly M Pickard JD Tshibanda L. Willful modulation of brain activity in disorders of consciousness. N Engl J Med. (2010) 362:579–89. 10.1056/NEJMoa0905370 20130250

[16] Schiff ND. Cognitive motor dissociation following severe brain injuries. JAMA Neurol. (2015) 72:1413. 10.1001/jamaneurol.2015.2899 26502348

[17] Estraneo A Moretta P Loreto V Lanzillo B Santoro L Trojano L. Late recovery after traumatic, anoxic, or hemorrhagic long-lasting vegetative state. Neurology (2010) 75:239–45. 10.1212/WNL.0b013e3181e8e8cc 20554941

[18] Luaute J Maucort-Boulch D Tell L Quelard F Sarraf T Iwaz J Long-term outcomes of chronic minimally conscious and vegetative states. Neurology (2010) 2010:75 10.1212/WNL.0b013e3181e8e8df 20554940

[19] Chan A-W Tetzlaff JM Altman DG Laupacis A Gøtzsche PC KrleŽa-Jerić K. SPIRIT 2013 Statement: defining standard protocol items for clinical trials. Ann Intern Med. (2013) 158:200. 10.7326/0003-4819-158-3-201302050-00583 23295957 PMC5114123

[20] Posner J Plum F Saper C Plum and Posner's Diagnosis of Stupor and Coma. New York, NY: Oxford University Press (2007).

[21] Kondziella D Fisher PM Larsen VA Hauerberg J Fabricius M Møller K. Functional MRI for assessment of the default mode network in acute brain injury. Neurocrit Care (2017) 27:401–6. 10.1007/s12028-017-0407-6 28484929

[22] Kirsch M Guldenmund P Ali Bahri M Demertzi A Baquero K Heine L. Sedation of patients with disorders of consciousness during neuroimaging: effects on resting state functional brain connectivity. Anesth Analg. (2017) 124:588–98. 10.1213/ANE.0000000000001721 27941576

[23] Rosanova M Gosseries O Casarotto S Boly M Casali AG Bruno M-A. Recovery of cortical effective connectivity and recovery of consciousness in vegetative patients. Brain (2012) 135:1308–20. 10.1093/brain/awr340 22226806 PMC3326248

[24] Liberati G Hünefeldt T Olivetti Belardinelli M. Questioning the dichotomy between vegetative state and minimally conscious state: a review of the statistical evidence. Front Hum Neurosci. (2014) 8:865. 10.3389/fnhum.2014.00865 25404905 PMC4217390

[25] Bruno M-A Vanhaudenhuyse A Thibaut A Moonen G Laureys S. From unresponsive wakefulness to minimally conscious PLUS and functional locked-in syndromes: recent advances in our understanding of disorders of consciousness. J Neurol. (2011) 258:1373–84. 10.1007/s00415-011-6114-x 21674197

[26] Giacino JT Katz DI Schiff ND Whyte J Ashman EJ Ashwal S. Practice guideline update recommendations summary: disorders of consciousness. Neurology (2018) 91:450–60. 10.1212/WNL.0000000000005926 30089618 PMC6139814

[27] Gerrard P Zafonte R Giacino JT. Coma Recovery Scale-Revised: evidentiary support for hierarchical grading of level of consciousness. Arch Phys Med Rehabil. (2014) 95:2335–41. 10.1016/j.apmr.2014.06.018 25010536

[28] Demertzi A Antonopoulos G Heine L Voss HU Crone JS de Los Angeles C Intrinsic functional connectivity differentiates minimally conscious from unresponsive patients. Brain (2015) 2015:138 10.1093/brain/awv169 26117367

[29] Whitfield-Gabrieli S Nieto-Castanon A. *Conn* : a functional connectivity toolbox for correlated and anticorrelated brain networks. Brain Connect (2012) 2:125–41. 10.1089/brain.2012.0073 22642651

[30] Behzadi Y Restom K Liau J Liu TT. A component based noise correction method (CompCor) for BOLD and perfusion based fMRI. Neuroimage (2007) 37:90–101. 10.1016/j.neuroimage.2007.04.042 17560126 PMC2214855

[31] Fisher PM Larsen CB Beliveau V Henningsson S Pinborg A Holst KK. Pharmacologically induced sex hormone fluctuation effects on resting-state functional connectivity in a risk model for depression: a randomized trial. Neuropsychopharmacology (2017) 42:446–53. 10.1038/npp.2016.208 27649641 PMC5399242

[32] André-Obadia N Zyss J Gavaret M Lefaucheur J-P Azabou E Boulogne S. Recommendations for the use of electroencephalography and evoked potentials in comatose patients. Neurophysiol Clin. (2018) 48:143–69. 10.1016/j.neucli.2018.05.038 29784540

[33] Azabou E Navarro V Kubis N Gavaret M Heming N Cariou A. Value and mechanisms of EEG reactivity in the prognosis of patients with impaired consciousness: a systematic review. Crit Care (2018) 22:184. 10.1186/s13054-018-2104-z 30071861 PMC6091014

[34] Holeckova I Fischer C Giard M-H Delpuech C Morlet D. Brain responses to a subject's own name uttered by a familiar voice. Brain Res. (2006) 1082:142–52. 10.1016/j.brainres.2006.01.089 16703673

[35] Synek VM. Prognostically important EEG coma patterns in diffuse anoxic and traumatic encephalopathies in adults. J Clin Neurophysiol. (1988) 5:161–74. 3074973 10.1097/00004691-198804000-00003

[36] Claassen J Velazquez A Meyers E Witsch J Falo MC Park S. Bedside quantitative electroencephalography improves assessment of consciousness in comatose subarachnoid hemorrhage patients. Ann Neurol. (2016) 80:541–53. 10.1002/ana.24752 27472071 PMC5042849

[37] Chennu S Annen J Wannez S Thibaut A Chatelle C Cassol H. Brain networks predict metabolism, diagnosis and prognosis at the bedside in disorders of consciousness. Brain (2017) 140:2120–32. 10.1093/brain/awx163 28666351

[38] Engemann DA Raimondo F King J-R Rohaut B Louppe G Faugeras F. Robust EEG-based cross-site and cross-protocol classification of states of consciousness. Brain (2018) 141:3179–92. 10.1093/brain/awy251 30285102

[39] Peinkhofer C Martens P Grand J Truelsen T Knudsen GM Kjaergaard J. Influence of strategic cortical infarctions on pupillary function. Front Neurol. (2018) 9:916. 10.3389/fneur.2018.00916 30420833 PMC6215832

[40] Stoll J Chatelle C Carter O Koch C Laureys S Einhäuser W. Pupil responses allow communication in locked-in syndrome patients. Curr Biol. (2013) 23:R647–8. 10.1016/j.cub.2013.06.011 23928079

[41] Giacino JT Kalmar K Whyte J. The JFK Coma Recovery Scale-Revised: measurement characteristics and diagnostic utility. Arch Phys Med Rehabil. (2004) 85:2020–9. 10.1016/j.apmr.2004.02.033 15605342

[42] Giacino JT Ashwal S Childs N Cranford R Jennett B Katz DI. The minimally conscious state: definition and diagnostic criteria. Neurology. (2002) 349–53. 10.1212/WNL.58.3.349 11839831

[43] Giacino JT Fins JJ Laureys S Schiff ND. Disorders of consciousness after acquired brain injury: the state of the science. Nat Rev Neurol. (2014) 10:99–114. 10.1038/nrneurol.2013.279 24468878

[44] Seel RT Sherer M Whyte J Katz DI Giacino JT Rosenbaum AM. Assessment scales for disorders of consciousness: evidence-based recommendations for clinical practice and research. Arch Phys Med Rehabil. (2010) 91:1795–813. 10.1016/j.apmr.2010.07.218 21112421

[45] Klipin M Mare I Hazelhurst S Kramer B. The process of installing REDCap, a web based database supporting biomedical research. Appl Clin Inform. (2014) 5:916–29. 10.4338/ACI-2014-06-CR-0054 25589907 PMC4287671

[46] Rohaut B Naccache L. What are the boundaries of unconscious semantic cognition? Eur J Neurosci. (2018) 47:1287–8. 10.1111/ejn.13930 29729224

[47] Edlow BL Chatelle C Spencer CA Chu CJ Bodien YG O'Connor KL. Early detection of consciousness in patients with acute severe traumatic brain injury. Brain (2017) 140:23–31. 10.1093/brain/awx176 29050383 PMC6059097

[48] Crick F Koch C. A framework for consciousness. Nat Neurosci. (2003) 6:119–26. 10.1038/nn0203-119 12555104

[49] Weijer C Bruni T Gofton T Young GB Norton L Peterson A. Ethical considerations in functional magnetic resonance imaging research in acutely comatose patients. Brain (2015) 139(Pt 1):292–9. 10.1093/brain/awv272 26373606 PMC5839553

